# Realization of Large-Area Wrinkle-Free Monolayer Graphene Films Transferred to Functional Substrates

**DOI:** 10.1038/srep09610

**Published:** 2015-06-05

**Authors:** Byeong-Ju Park, Jin-Seok Choi, Hyun-Suk Kim, Hyun-You Kim, Jong-Ryul Jeong, Hyung-Jin Choi, Hyun-June Jung, Min-Wook Jung, Ki-Seok An, Soon-Gil Yoon

**Affiliations:** 1 Department of Materials Engineering, Chungnam National University, Daeduk Science Town, 305-764, Daejeon, Korea; 2 Research Analysis Center, Korea Advanced Institute of Science and Technology, 291 Daehak-ro, Yuseong-gu, 305-701, Daejeon, Korea; 3 Department of Nanomaterials Engineering, Chungnam National University, Daeduk Science Town, 305-764, Daejeon, Korea; 4 Thin Film Materials Research Group, Korea Research Institute of Chemical Technology, 19 Sinseongno, Yuseong-gu, Daejeon 305–600, Korea

## Abstract

Structural inhomogeneities, such as the wrinkles and ripples within a graphene film after transferring the free-standing graphene layer to a functional substrate, degrade the physical and electrical properties of the corresponding electronic devices. Here, we introduced titanium as a superior adhesion layer for fabricating wrinkle-free graphene films that is highly applicable to flexible and transparent electronic devices. The Ti layer does not influence the electronic performance of the functional substrates. Experimental and theoretical investigations confirm that the strong chemical interactions between Ti and any oxygen atoms unintentionally introduced on/within the graphene are responsible for forming the clean, defect-free graphene layer. Our results accelerate the practical application of graphene-related electronic devices with enhanced functionality. The large-area monolayer graphenes were prepared by a simple attachment of the Ti layer with the multi-layer wrinkle-free graphene films. For the first time, the graphene films were addressed for applications of superior bottom electrode for flexible capacitors instead of the novel metals.

Since its initial preparation by mechanical exfoliation, the chemical vapor deposition (CVD) of graphene onto Ni^1^, Cu^2^,^3^,^4^,^5^, and hydrogen-terminated Ge substrates^6^ has been regarded as the most reasonable large-scale method to synthesize graphene. Wafer-scale graphene films are potential field-effect transistors (FETs)^7^,^8^,^9^,^10^ and transparent conducting layers^11^,^12^,^13^,^14^, especially for flexible/stretchable electronics. However, structural inhomogeneities, such as wrinkles and ripples, form within a graphene film upon the transfer of a free-standing graphene layer to various functional substrates, which degrades the physical and electrical properties of the corresponding electronic devices^15^,^16^,^17^,^18^,^19^. In addition, the oxygen atoms that unavoidably contaminate the transferred graphene films may contribute to such degradation^20^,^21^.

Platinum (Pt) is commonly used as the bottom electrode in thin-film devices due to its high work function (5.12–5.93 eV) and excellent chemical stability at high temperatures. A titanium (Ti) layer is generally used to improve the adhesion between Pt and oxygen-containing or oxygen-philic substrates, such as Si (with native oxide), SiO_2_/Si, glass, and polymers^22^,^23^,^24^. However, the exact function of the Ti adhesion layer is still unclear.

Here, we hypothesize that Ti interacts with the oxygen in the substrates to form strong Ti-O chemical bonds and utilize this concept to transfer wrinkle-free graphene films to various functional substrates by introducing a Ti adhesion layer. We demonstrate that defect-free graphene films on Ti adhesion layers can be used as transparent, reliable bottom electrodes without degrading the performance, even with mechanical bending, unlike conventional Pt electrodes.

To gain fundamental insights into the excellent adhesion between Ti layers and oxygen-containing or oxygen-philic substrates, we deposited a 50 nm-thick Ti film on a glass substrate and observed the chemical nature at their interface via X-ray photoelectron spectroscopy (XPS) after an exposure at an air atmosphere. Various TiO_x_ phases such as TiO, TiO_2_, Ti_2_O_3_, and Ti_3_O_5_ as well as metallic Ti were found at the Ti-SiO_2_ (main phase of glass) interface, suggesting that a large portion of the Ti interacted chemically with oxygen (oxidized), which might be from the glass substrate (Figs. 1a and 1b). The surface states of the Ti layer (Fig. 1c) were consisted of the TiO_x_ phases such as TiO_2_, Ti_3_O_5_, and Ti because as-deposited Ti surface layer was exposed at an air atmosphere. These TiO_x_ phases were also observed at a SiO_2_-coated Si interface (Supplementary Figs. 1a and 1b). Although the Ti adhesion layers were deposited onto the glass substrates, the Ti adhesion layer (below 10 nm thickness) deposited onto the glass substrate did not influence the transmittance of the glass substrate, as shown in Fig. 1(d). In addition, the various thick-Ti adhesion layers showed comparable resistivity to that of the glass substrate (see Fig. 1(e)). These results suggested that the Ti adhesion layers did not influence the performance of the electronic devices prepared on the glass substrate. In here, thickness of the Ti adhesion layer was limited below 10 nm to ensure the performance of the electronic devices. Subsequent mechanical tests on the synthesized samples confirmed that the TiO_x_ phases cause the robust Ti-substrate interface (Supplementary Fig. 2).

Chemically synthesized graphene films are inevitably exposed to oxygen-rich conditions during post processing via physical or chemical treatments. Therefore, the final graphene products incorporate oxygen species^20^,^21^,^25^. Hong^20^ and Kim^21^ et al. reported that carbon and oxygen bonding states existed in the graphene films after the transfer step. Mkhoyan et al.^25^ also reported that the oxygen atoms were randomly attached to carbon atoms on both sides of the graphene sheet based on scanning transmission electron microscopy combined with electron energy loss spectroscopy (EELS).

In these contexts, we used the Ti layer as a binding layer between the graphene film and a transferred substrate. Naturally existing oxygen species at the graphene film surface provide the oxygen for the formation of strong Ti-O chemical bonds, which benefits the adhesion between graphene and the substrate.

The two-dimensional atomic force microscopy (AFM) images for a graphene layer transferred to a glass substrate without a Ti adhesion layer showed severe wrinkling (Fig. 2b), which indicates a high root-mean-square (rms) roughness. The graphene wrinkles disappeared upon the addition of a Ti adhesion layer to yield wrinkle-free graphene (Figs. 2d and 2e). The rms roughness for graphene transferred to the glass substrate without a Ti layer was approximately 4.5 nm, while the Ti adhesion layer decreased this roughness (Fig. 2a). The rms graphene roughness was maintained at approximately 0.2 ± 0.03 nm using a 10 nm-thick-Ti layer and increased to 0.2–0.3 nm when the Ti thickness was increased to above 10 nm. It was also noted that the rms roughness of the bare-Ti layer as a function of thickness increased slightly from 0.2 to 0.3 ± 0.04 nm (Fig. 2a). For evidence of the wrinkle-free characteristics in large areas using AFM, Figures 2(f and 2(g)) showed the AFM image observed at 1 × 1 and 5 × 5 μm^2^ using graphene films transferred to the 10 nm-thick Ti layers, respectively. Compared with the image observed at a small area (Fig. 2(f)), graphene films observed at a large area also showed no wrinkles, as shown in Fig. 2(g), presenting a rms roughness of 0.21 ± 0.03 nm. In contrast, a graphene layer transferred to a fully oxidized TiO_2_ layer grown on a glass substrate via atomic layer deposition had roughness as high as approximately 3.5–4.0 nm, (Supplementary Fig. 3), which is similar to graphene transferred to bare-glass without a Ti adhesion layer (Fig. 2a). Our findings revealed that the formation of the -Ti-O-C- bonds with the TiO_x_ is required to form wrinkle- and defect-free graphene films.

We used density functional theory (DFT) calculations and studied the Ti-graphene interface morphology to determine the role the Ti–O chemical bonds and under-coordinated Ti sites play in forming a wrinkle-free graphene layer on the Ti adhesion layer (Fig. 3a). The DFT-predicted Ti and graphene-oxide film interface essentially confirmed the experimental findings. After a graphene oxide layer binds to the Ti (0001) surface, the Ti atoms draw oxygen from the graphene oxide to form Ti–O networks. Moreover, some of the under-coordinated Ti atoms provide space to form –Ti–O–C–. The central oxygen atoms bridge the Ti and graphene which allows a graphene layer to lie above the Ti–O networks without wrinkling. The maximum displacement of the carbon atoms along the normal axis is as low as 0.086 nm (Fig. 3b).

The experimental and theoretical findings for the chemical nature of the graphene-Ti (or TiO_x_) interface were confirmed via composition sensitive annual dark field (ADF) imaging and electron energy loss spectroscopy (EELS) mapping. The transferred graphene films consisted of approximately 5-6 layers in the ADF image (Fig. 3c), and image mapping at 0.1 nm intervals across a selected area in the ADF image (yellow rectangle of upper inset) revealed the presence of carbon (Fig. 3d), TiO_x_ (Fig. 3e), and oxygen (Fig. 3f), whereas pure Ti was not observed. The EELS mapping at the interface between the graphene and Ti adhesion layer showed a clear region of the color change between the graphene and TiO_x_ within a range of 1.0–2.0 nm (dotted region in Figs. 3g and 3h). If the bonding between the graphene and the TiO_x_ is not formed, the proper colors of the graphene and TiO_x_ only existed at an interface. As a result, the typical color changes at an interface were distinctly attributed to the chemical bonding between the graphene films and the TiO_x_ of the adhesion layer. The core-loss EELS spectra of (Supplementary Fig. 4b) the C K-edge and (Supplementary Fig. 4c) Ti L-edge from the selected area (Supplementary Fig. 4a) also confirms the presence of the –Ti–O–C– chemical bonds at the graphene and Ti adhesion layer interface.

The bonding energy (282 eV) of the Ti-C(O) is higher than that of the C-C bonding (Van de Waals bonding: 60 meV) between the layers of the graphene. Accordingly, Ti adhesion layer coated on the glass substrate may strongly bind with the first layer of the graphene by a mechanical attachment. If this concept is possibly realized, actual monolayer graphene can be obtained from the multi-layer graphenes transferred to the substrates as well as an additional demonstration for a strong adhesion between Ti layers with the graphene. The preparation of large-area monolayer graphene from the wrinkle-free multi-layer graphene films transferred to the Ti (10 nm)/glass substrate was performed using the method shown in an inset of Fig. 4(a). Figure 4(a) showed the Raman spectra of the bare- and top-graphene films before and after mechanical detachment. Here, the bare-graphene means the wrinkle-free multi-layer graphenes transferred to the Ti/glass. The “top” sample showed the clear Raman spectrum exhibiting the representative graphene films, as compared with that of bare-graphene films. Based on the Raman spectrum of the “top” sample, Ti adhesion layer by a slight mechanical attachment only induced a strong adhesion with the graphene films, producing a monolayer graphene from the multi-layers. The quality of the monolayer graphenes prepared newly by the Ti adhesion layer should be developed further. Figure 4(b) showed the transmittance of the bare- and top- graphenes. The bare-graphenes were consisted of 5–6 layers, as shown in Fig. 3(c). Transmittance of the bare- and top-graphenes was approximately 87 and 96% at a wavelength of 550 nm, respectively. The actual monolayer graphene was reported to be exhibited a transmittance of approximately 96–97% at a wavelength of 550 nm^26^. The transmittance using UV-Vis spectrometer was measured at a diameter of 0.8 cm, meaning a large-area. The result of transmittance suggested that the top-graphene was an actual monolayer. Based on this process, many monolayer graphenes with a large size were easily obtained by a mechanical attachment after a transferal of multi-layer graphene films to the Ti adhesion layer.

Wrinkle-free graphene films on a Ti adhesion layer were addressed for the bottom electrode applications in transparent and flexible thin film capacitors. For thin film capacitors, Bi_2_Mg_2/3_Nb_4/3_O_7_ (BMNO) dielectric materials with pyrochlore structures were chosen to provide a high dielectric permittivity in amorphous films grown at room temperature, which is critical for flexible devices^27^. Our 200 nm-thick BMNO films deposited at room temperature exhibited a leakage current density of approximately 10^−8^ A/cm^2^ at 10 V and a dielectric permittivity of approximately 40–60 at 100 kHz^28^,^29^,^30^. The measured rms roughness of BMNO (200 nm)/graphene/Ti/glass thin films with different Ti layer thicknesses represented the rms roughness trend for graphene/glass as a function of Ti thickness (Supplementary Fig. 5a). Moreover, the surface morphology of a Pt top electrode improved dramatically with an embedded Ti layer (10 nm-thick), which ensures the device reliability (Supplementary Figs. 5b and 5c).

The dielectric constants for the studied capacitors were between 44 and 46 at a frequency of 100 kHz, which represents a slight dielectric dispersion with increasing frequency (Fig. 4c). The capacitors without a Ti adhesion layer mostly failed during the dielectric property measurements. However, the dielectric properties for capacitors containing a Ti adhesion layer were reliably measured in more than 95% of the samples. Compared to the dielectric loss (dissipation factor) for capacitors with a Pt bottom electrode, the dissipation factor of films grown on the graphene/Ti (2, 10 nm) increased at 2–3 × 10^5^ Hz because of the high resistivity (~10^−4^ Ω-cm) of graphene. Using oxide electrodes, such as indium and Al-doped ZnO (AIZO)^31^, LaNiO_3_^32^, and La_0.5_Sr_0.5_CoO_3_ (LSCO)^33^, increased the dielectric loss for frequency regions above 10^5^ Hz due to their higher resistivity (~10^−4^ Ω-cm) relative to the metals (ρ = 1–10 μΩ-cm in the case of Pt, Au etc.)^34^. The increased dielectric loss at higher frequencies was attributed to adding the electrode portion to the dielectric loss of the capacitor. The BMNO capacitors without a Ti adhesion layer exhibited a high leakage current density (approximately 10 A/cm^2^) using an applied voltage of ±5 V (Fig. 4d). However, the capacitors with a Ti adhesion layer showed improved leakage properties, particularly for the leakage current density of capacitors with 10 nm-thick Ti adhesion layers (approximately 8 × 10^−8^ A/cm^2^ at ±5 V), which approached those of conventional capacitors with a Pt/Ti bottom electrode. The capacitors containing the Ti adhesion layers exhibited stable electrical properties (approximately 3 × 10^−7^ A/cm^2^) even at a voltage range of ±20 V (Fig. 4e). Wrinkle-free graphene electrodes containing a Ti adhesion layer are reliable bottom electrodes comparable to their conventional Pt electrode counterparts. To be used as flexible capacitors, graphene capacitors built on a flexible substrate should be stable with no severe degradation in electrical properties upon the application of mechanical stress. The AFM images for the graphene/Ti (10 nm)/flexible-PET (polyethylene terephthalate) substrate showed no severe structural defects after bending, and the corresponding rms roughness values were consistent within the error range (Supplementary Fig. 6). Despite being inserted between the graphene and PET substrate, the 10 nm-thick Ti layer did not affect the bendability of the graphene. The leakage current density measured after the bending test (0.6 cm for 30 s, Fig. 4f) confirmed that our BMNO/graphene/Ti (10 nm)/PET was mechanically stable without any electrical property degradation. For long-term mechanical stability of the graphene/Ti (10 nm)/PET, the leakage current densities of the BMNO/graphene/Ti/PET were investigated after ten times bending using the same samples. Their results were included in Fig. 4 (f). The leakage current densities of the graphene-capacitors after various times bending were not changed, as compared with that of the first bending. This result suggested that the graphene transferred to the Ti layer showed a long-term mechanical stability for the transparent electronics application. In contrast, conventional Pt/Ti electrode capacitors exhibited an abrupt increase in their leakage current density upon bending because of the non-flexible Pt layer. Based on their structural and electrical properties after bending, we suggest that graphene transferred to a Ti adhesion layer is a superior bottom electrode for flexible capacitors. The Ti adhesion layer plays an essential role in flexible and transparent capacitors using graphene electrodes.

## Methods

### Characterization of the Ti adhesion layer

In order to investigate the role of the adhesion layer of the Ti films, 50 nm-thick Ti thin films were deposited onto the glass and SiO_2_ (250 nm)/Si substrates using dc sputtering under argon atmosphere at room temperature with a 2 inch Ti metal target (purity: 99.99%). DC power for the thin film deposition was maintained at 20 W. Thin film morphologies were characterized using atomic force microscopy (AFM, Auto Probe CP) and field-emission scanning electron microscope ((FE-SEM, Sirion, FEI (Netherlands)). The interface of the Ti substrates has been investigated using X-ray photoelectron spectroscopy (XPS, Thermoscientific K-Alpha). To rationalized the effect of oxygen involved in the substrate, the adhesion test of the Ti layer deposited onto the oxide layer (SiO_2_/Si) and non-oxide layer (CrN/Si) was performed by the scratch test using a scratch tester (PVD-coatings Co., UK), and the amount of the residual Ti in the scratched regions was measured using energy dispersive spectroscopy (EDS).

### Wrinkle-free graphene films transferred to the Ti adhesion layer

Graphene films were grown at 900°C onto the Ni (250 nm)/Si substrate via rapid-thermal pulse chemical vapor deposition (RTP CVD, NCD Tech. Co. Korea) and an aqueous iron chloride (1 M FeCl_3_) solution was used as an oxidizing etchant to remove the nickel layers^35^. For the stronger adhesion, the graphene transferred to the Ti adhesion layers deposited onto the glass and PET substrate was heated for 20 min at 60°C after rinsing using the diluted ionized water. The actual thickness of the Ti adhesion layer was measured via TEM cross-sectional image. Thicknesses of the Ti adhesion layers for transferral of the graphene were varied from 2 to 20 nm. The surface images and root-mean square (rms) roughness of the graphenes transferred onto the substrates were observed using AFM. The transmission and the sheet resistance of the Ti adhesion layers deposited onto the glass substrate were measured using the HP8453 UV-Vis spectrophotometer and the four-point probe method, respectively. The actual bonding between graphene and the Ti adhesion layer was investigated using XPS and EELS (GATAN, Quantum 965) attached at TEM.

### Preparation of the new graphene films from the wrinkle-free graphene films

**Figure 4:**
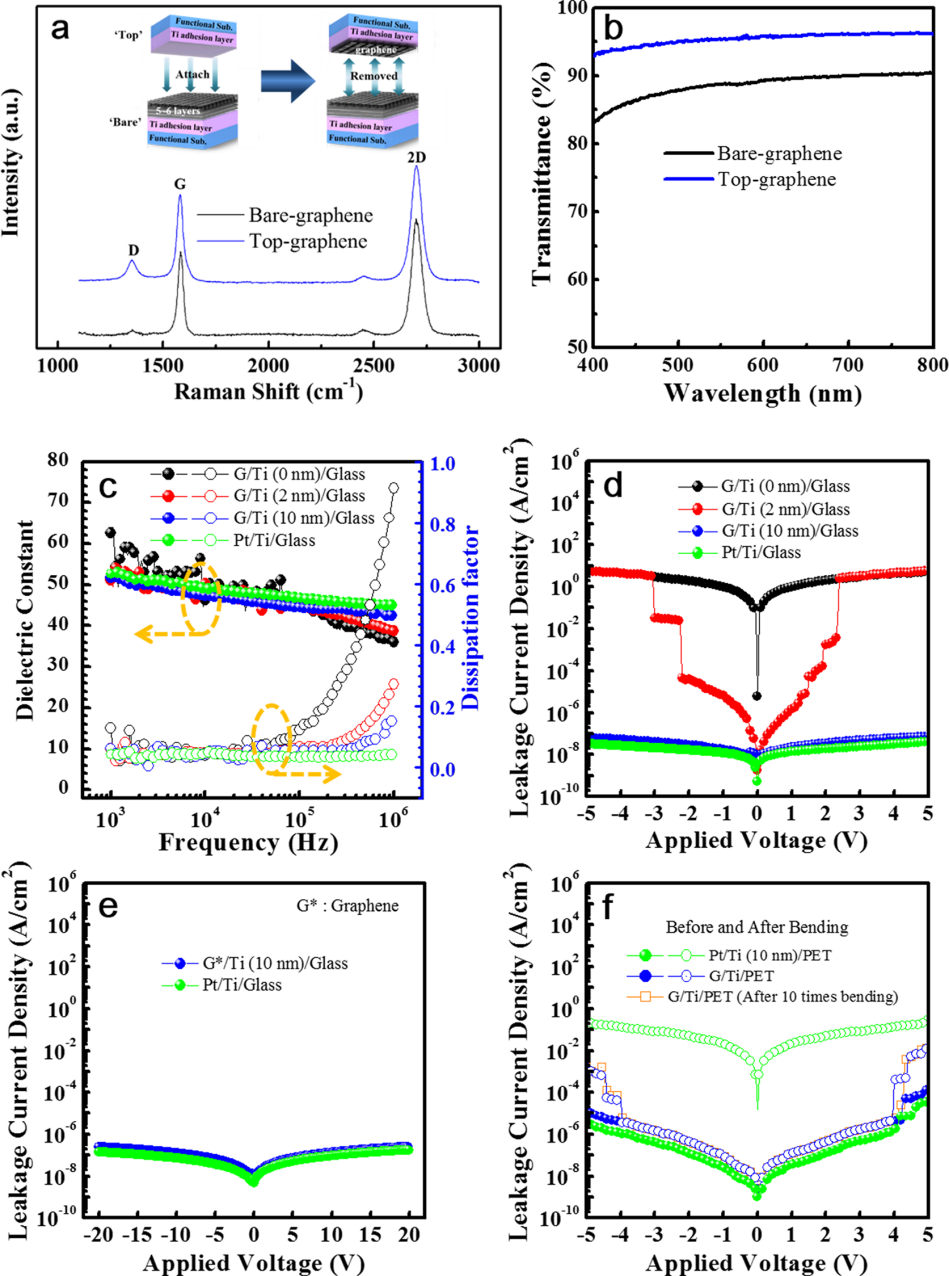
(a), Raman spectra of bare- and “top-” graphenes observed after a detachment of the Ti/glass substrate mechanically attached to the multi-layer graphene films transferred to the Ti/glass substrate. An inset of (a) showed a schematic structure to prepare the actual monolayer graphene from the multi-layer graphene films. (b), Transmittance of the bare- and “top-” graphenes observed after a mechanical detachment of the Ti/glass substrate. (c), Dielectric constants and dissipation factors for capacitors with Ti adhesion layers of 0, 2, and 10 nm as a function of the applied frequency. (d), The leakage current densities for capacitors with Ti adhesion layers of 0, 2 and 10 nm as a function of the applied voltage. (e), BMNO/graphene/Ti (10 nm)/glass capacitors showing a stable leakage current density of approximately 3 × 10^−7^ A/cm^2^ for an applied voltage of ±20 V. (f), Leakage current densities vs. the applied voltage for capacitors using graphene films transferred to the 10 nm-thick Ti adhesion layer coated on PET before (close circle) and after (open circle) bending at 0.6 cm for 30 s. Here, leakage current densities measured after ten times bending using the same samples were also shown in (f) for long-term mechanical stability of the graphene/Ti/PET. Dielectric and leakage currents for the BMNO/graphene/Ti/glass or PET capacitors were compared to those of the BMNO/Pt/Ti/glass or PET capacitors. Thickness of the Pt top and bottom electrodes was approximately 150 nm, respectively.

The new Ti/glass substrates (top sample) were mechanically putted on the multi-layer graphene films/Ti/glass (bottom sample) in a vacuum atmosphere to prevent the oxidation of the Ti layer deposited on the glass substrate. Here, the Ti layer in a new Ti/glass substrate was attached to the graphene films transferred to the Ti/glass substrate. The schematic structure for the preparation of the new graphene films from the wrinkle-free graphene films was inserted at Fig. 4(a). After maintaining at room temperature for a constant time of approximately 1 h, top sample (Ti/glass) was mechanically detached from the bottom sample. The graphene films prepared newly from the graphene films transferred to the Ti/glass substrate were characterized using Raman and HP8453 UV-Vis spectrophotometer.

### Transparent and flexible thin film capacitor using Bi_2_Mg_2/3_Nb_4/3_O_7_ (BMNO)/graphene/Ti layers/glass or PET substrate

200 nm-thick BMNO high dielectric constant thin films were deposited at room temperature under an oxygen atmosphere via pulsed laser deposition (PLD) using a BMNO target (purity of 99.99%) with a diameter of one in. onto the graphene/Ti/glass (700 μm, Corning Gorilla) and PET (200 μm) substrates. The dielectric properties of the BMNO thin films were evaluated as a function of frequency using an impedance gain phase analyzer (HP4194A). The leakage current characteristics of the BMNO capacitors were investigated via HP4145B semiconductor parameter analysis. Bending tests of the graphene/Ti (10 nm)/PET and the 200 nm-thick BMNO films/graphene/Ti (2, 10 nm)/PET were performed by the method shown in the inset of fig. S6. One side of a 1 × 1 cm^2^ sample was fixed and the other side was pushed for 30 s at each point. Variations in rms roughness of the samples after bending at each point were measured by AFM. The 150 nm-thick Ni top electrodes (100 × 100 μm^2^ size) were deposited for the electrical measurement after the bending of the BMNO/graphene/Ti/PET.

### Computational Details

We performed GGA-level spin-polarized DFT calculations with the VASP code^36^ and the PBE functional^37^. The interaction between the ionic core and the valence electrons was described by the projector augmented wave method^38^, and the valence electrons were treated with a plane wave basis up to an energy cutoff 400 eV. The Brillouin zone was sampled at the Γ-point. The convergence criteria for the electronic structure and the geometry were 10^−4^ eV and 0.01 eV/Å, respectively. We used the Gaussian smearing method with a finite temperature width of 0.1 eV in order to improve convergence of states near the Fermi level. A single layer graphene with 72 carbon atoms and a 5 × 5 Ti (0001) slab with three Ti layers was used to describe a graphene film and a Ti adhesion layer, respectively. The bottom Ti layer was fixed during structural optimization whereas the others were released. Initially, a graphene film was oxidized with 25 oxygen atoms to produce a graphene oxide layer and the graphene-oxide film was subsequently attached to the Ti slab. Seven more oxygen atoms were appropriately added to the Graphene-Ti interface to fill out the Ti vacancies formed upon the graphene-Ti interaction.

## Supplementary Material

Supplementary Information Realization of Large-Area Wrinkle-Free Monolayer Graphene Films Transferred to Functional Substrates

## Figures and Tables

**Figure 1:**
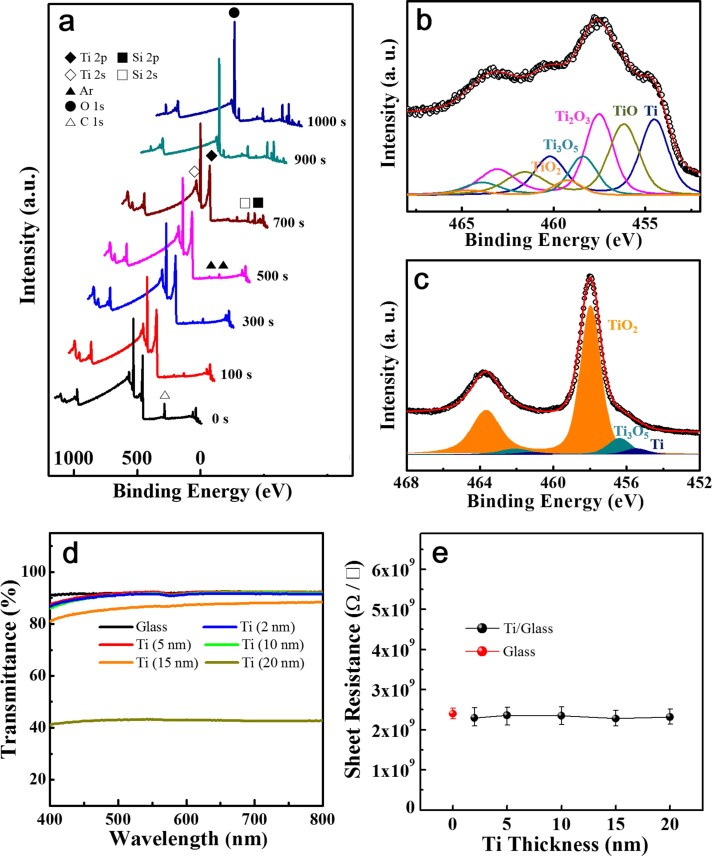
(a) XPS survey spectra as a function of etching time using a 50 nm-thick Ti layer grown on a glass substrate. The Ti layer was deposited at room temperature via direct current sputtering. (b) Curve fittings for the Ti 2p core level observed at an interface between Ti layer and glass substrate in a sample etched at 700 s. The silicon peak observed in the samples etched for 700 s was attributed to SiO_2_, which is the main phase of the glass. Etching for 700 s provided the interface between the Ti layer and glass substrate. (c) Curve fittings for the Ti 2p core level observed at a surface of Ti layer after an exposure in air atmosphere. (d) Transmittance of the Ti (2, 5, 10, 15, and 20 nm) adhesion layers deposited onto a glass substrate. (e), Sheet resistance of different Ti adhesion layer thicknesses. Sheet resistance of the glass substrate was indicated at (e).

**Figure 2:**
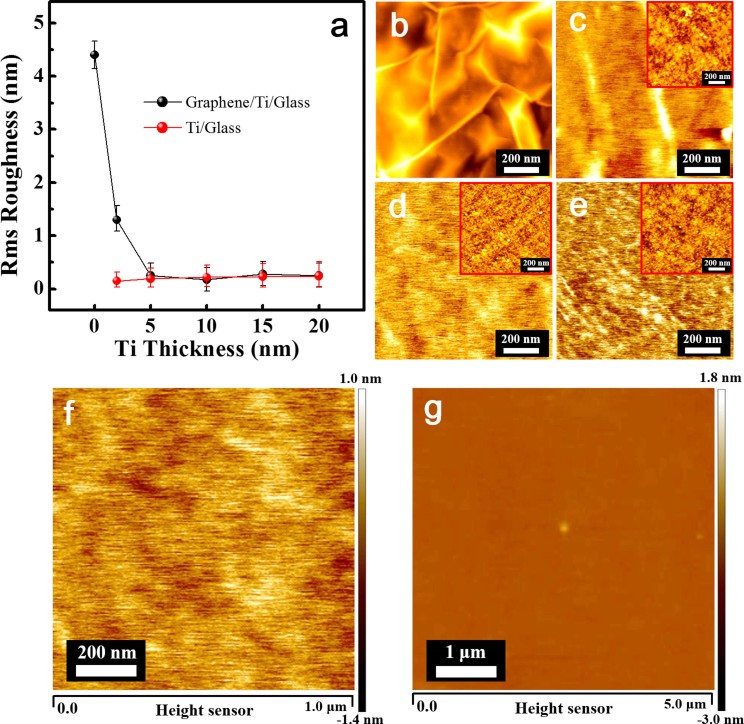
(a), The rms roughness for the graphene films transferred to Ti adhesion layers with various thicknesses. The variations in the rms roughness of the Ti/glass as a function of the Ti thickness are shown in (a). Two-dimensional AFM images (1 × 1 μm^2^) of the graphene films transferred to (b) 0, (c) 2, (d) 10, and (e) 20 nm-thick Ti adhesion layers. The upper insets in (c), (d), and (e) show the two-dimensional AFM images for Ti adhesion layers grown on the glass substrate. (f and g) Two-dimensional AFM images observed at 1 × 1 and 5 × 5 μm^2^ areas for the graphene films transferred to the 10 nm-thick Ti layer, respectively. The rms roughness of the (f) and (g) was approximately 0.17 ± 0.02 nm and 0.21 ± 0.03 nm, respectively.

**Figure 3:**
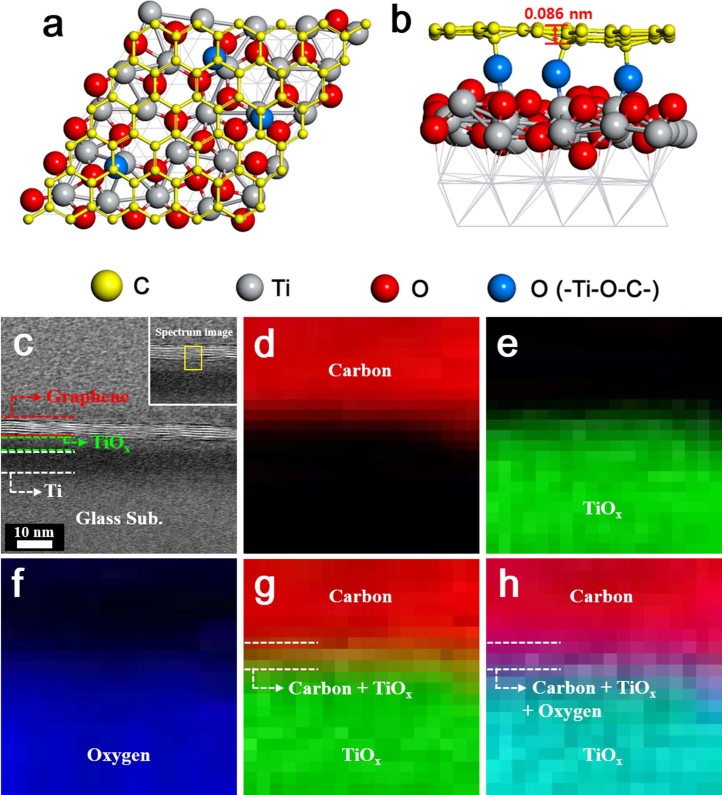
(a, b), The DFT-calculated morphologies at the interface; (c) the ADF image observed for the graphene film transferred to the Ti (10 nm)/glass. (d, e, and f), The EELS mapping images for the carbon (red), TiO_x_ (green), and oxygen (blue), respectively, observed in the selected area (yellow area in upper inset of (c). EELS mapping image observed at the bonding state of (g) (carbon and TiO_x_) and (h) (carbon, TiO_x_, and oxygen). The mapping results (color changes, dotted layer) shown in (g) and (h) indicate the chemical bonding between the graphene and Ti layer.
