# Supplementary material for: Realization of Large-Area Wrinkle-Free Monolayer Graphene Films Transferred to Functional Substrates
Source: Sci Rep. 2015 Jun 5;5:9610. doi: 10.1038/srep09610 (PMC4456724; doi:10.1038/srep09610)
Supplement: Supplementary Information — Realization of Large-Area Wrinkle-Free Monolayer Graphene Films Transferred to Functional Substrates [file srep09610-s1.pdf]

Supporting Information

Realization of Large-Area Wrinkle-Free Monolayer Graphene Films Transferred to Functional Substrates

Byeong-Ju Park[1], Jin-Seok Choi[1,2], Hyun-Suk Kim[1], Hyun-You Kim[3], Jong-Ryul Jeong,[1] Hyung-Jin Choi[1], Hyun-June Jung[1], Min-Wook Jung[4], Ki-Seok An[4], and Soon-Gil Yoon[1,*]

*Correspondence to sgyoon@cnu.ac.kr

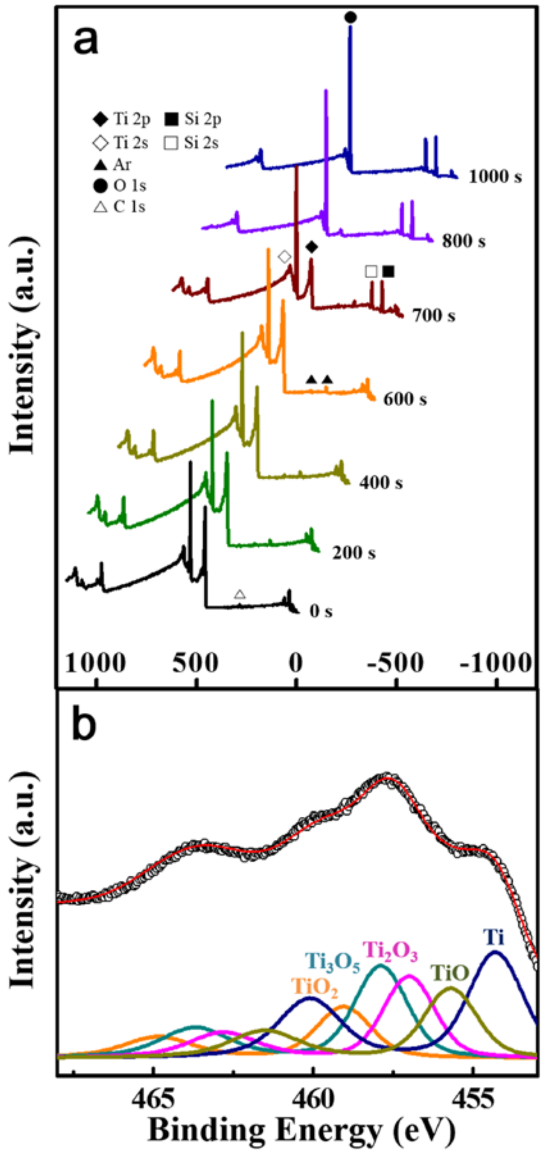

Figure 1 **Chemical bonding by a chemical reaction between a Ti adhesion layer and the oxygen that originated from the SiO_2 (250 nm)/Si (001) substrates. a,** XPS survey spectra observed as a function of etching time using a 50 nm-thick Ti layer grown onto a SiO_2/Si substrate. **b,** Curve fittings of the Ti 2p core level observed at the interface between a Ti layer and a SiO_2/Si substrate in a sample etched at 700 s.

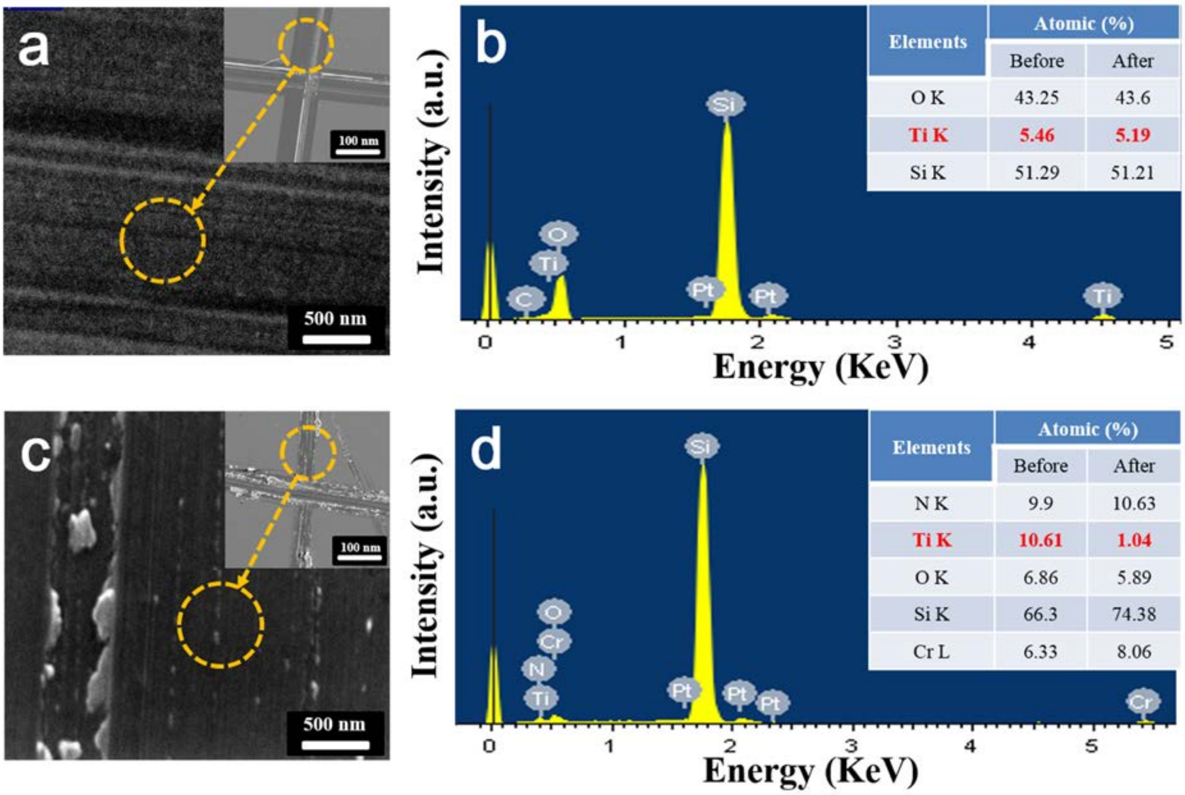

Figure 2 **Spectroscopic analyses of the scratched regions using Ti (50 nm)/SiO_2 (250 nm)/Si and Ti (50 nm)/CrN (100 nm)/SiO_2 (250 nm)/Si substrates. a,** SEM image and **b,** EDS elemental peaks of the scratched regions using a 50 nm-thick Ti layer grown onto a SiO_2 (250 nm)/Si substrate. **c,** SEM image and **d,** EDS elemental peaks of the scratched regions using the 50 nm-thick Ti layer grown

onto a CrN (100 nm)/SiO_2 (250 nm)/Si (001) substrate. Upper insets of the **(a)** and **(c)** showed the surface image of the scratched samples, respectively. The Pt peaks in EDS spectra were attributed to the Pt films deposited on the films for EDS analysis.

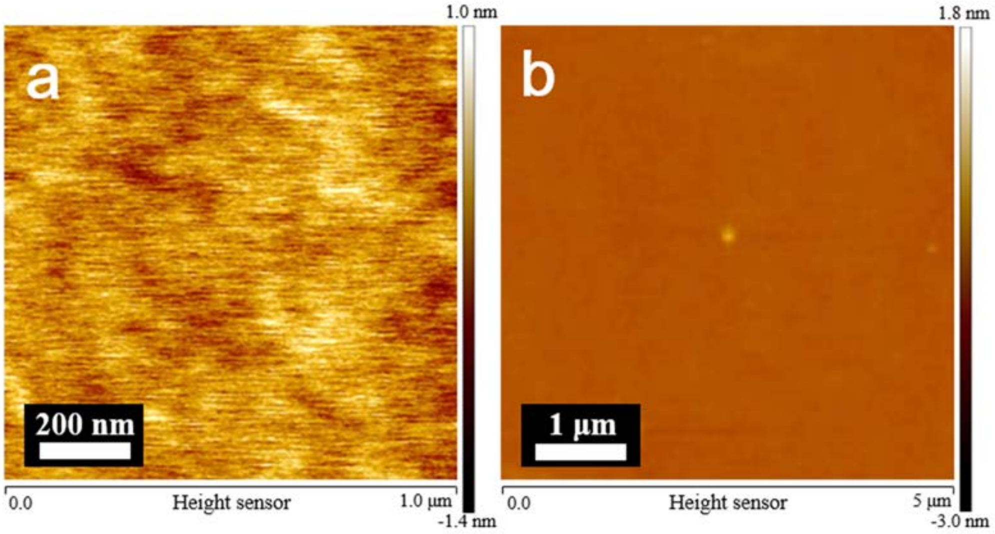

Figure 3 **AFM images of the TiO_2 (10 nm) film deposited onto the SiO_2 (250 nm)/Si (001) substrate at 160 °C using ALD and of the graphene transferred to the TiO_2 film. a, b,** Two-dimensional AFM images of the TiO_2 film and graphene transferred to the TiO_2 film, respectively. The rms roughness of the **(a)** and **(b)** was approximately 0.5 and 3.5-4.0 nm, respectively.

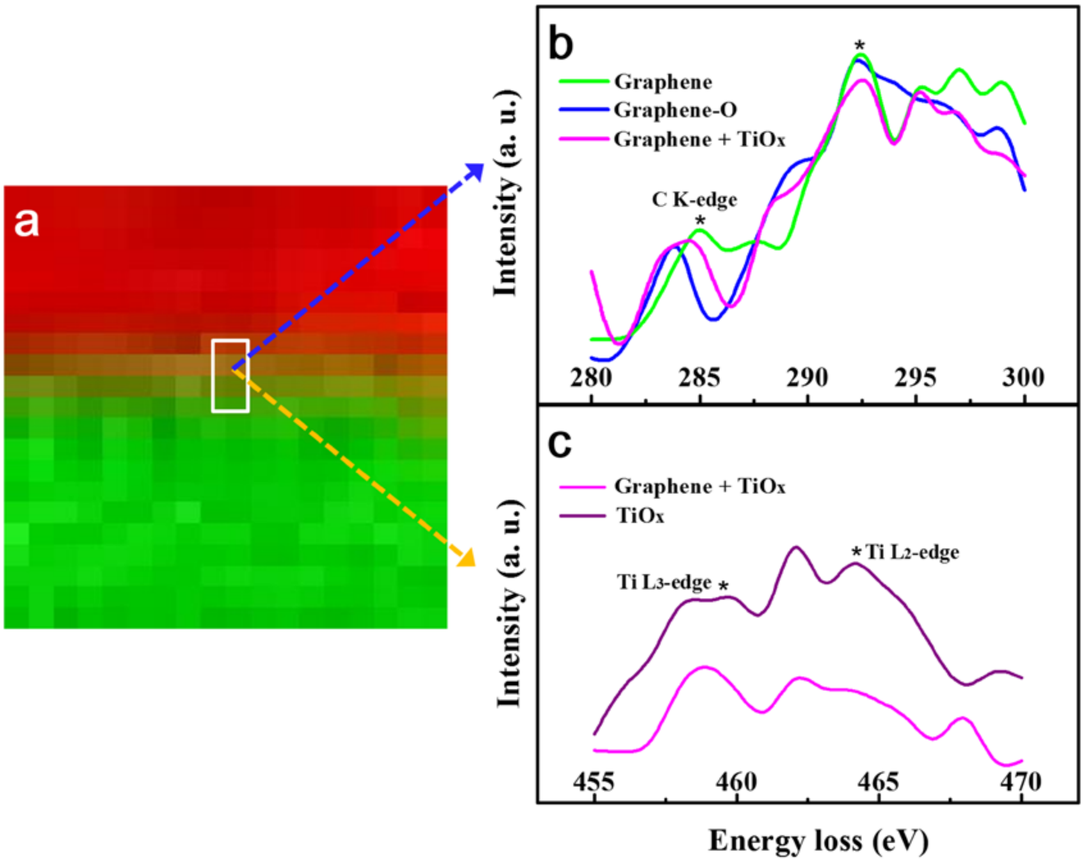

Figure 4 Electronic structure of graphene films transferred to the Ti adhesion layer. **a**, ADF image and Core-loss EELS spectra of **b**, C K-edge and **c**, Ti L-edge observed at a selected area (white color in (**a**)). The C K-edges for graphene, graphene-O, and bonding of the graphene and TiO_x were clearly shown in (**b**). The Ti L-edges for bonding of the graphene + TiO_x and TiO_x were shown in (**c**).

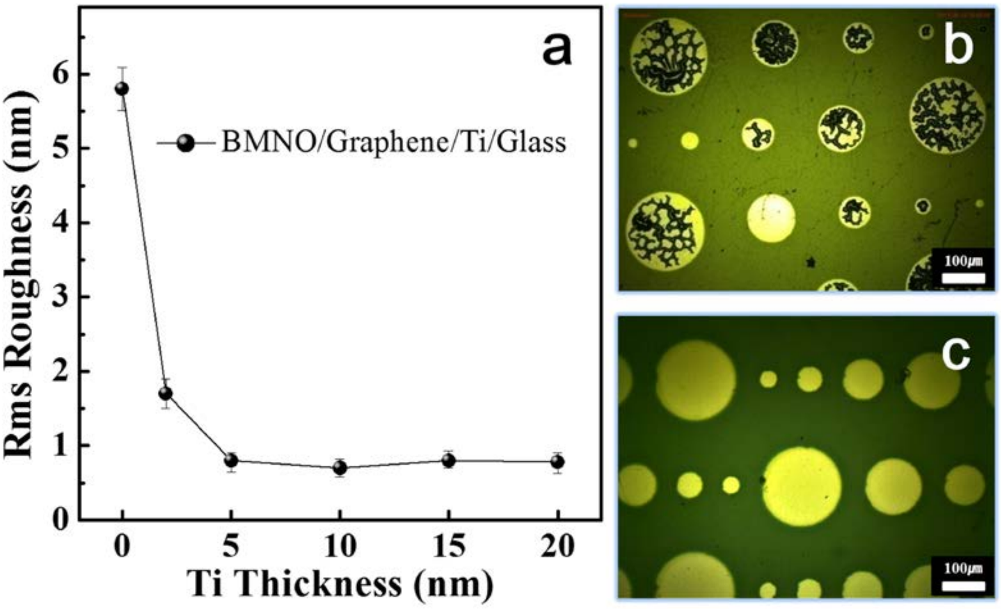

Figure 5 **Various spectroscopic analyses of the BMNO/graphene/Ti adhesion layer/glass**. **a,** The rms roughness of the 200 nm-thick BMNO films grown onto the graphene films transferred to the various Ti adhesion layer thicknesses. **b, c,** Optical images of the Pt top electrodes of various sizes deposited on the BMNO/graphene/glass and BMNO/graphene/Ti (10 nm) adhesion layer/glass, respectively. The Pt top electrode was severely damaged in the case of the absence of the Ti layer (**b**). For Pt/BMNO/graphene/Ti (10 nm)/glass capacitors, the Pt top electrode (**c**) showed a smooth morphology, which was suitable for measurement of the electrical properties.

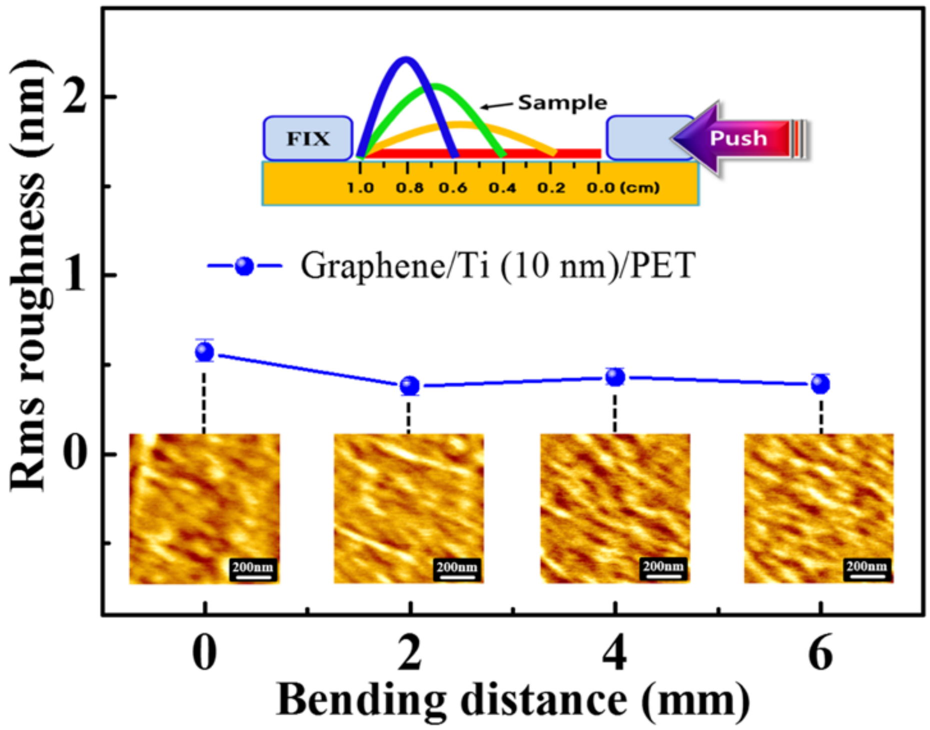

Figure 6 **Bendability of the graphene transferred to the Ti adhesion layer coated PET flexible substrates**. Variations in rms roughness observed after bending for 30 s at each point using the graphene films transferred to the 10 nm-thick Ti adhesion layer coated PET substrate. Upper inset showed a schematic diagram for the bending test, and lower insets showed two-dimensional AFM images observed at each point.
